# Inflammatory Bowel Disease: A Comprehensive Analysis of Molecular Bases, Predictive Biomarkers, Diagnostic Methods, and Therapeutic Options

**DOI:** 10.3390/ijms25137062

**Published:** 2024-06-27

**Authors:** Eguzkiñe Diez-Martin, Leidi Hernandez-Suarez, Carmen Muñoz-Villafranca, Leire Martin-Souto, Egoitz Astigarraga, Andoni Ramirez-Garcia, Gabriel Barreda-Gómez

**Affiliations:** 1 Research and Development Department, IMG Pharma Biotech S.L., 48170 Zamudio, Spain; eguz@imgpharma.com (E.D.-M.); leidi@imgpharma.com (L.H.-S.);; 2 Department of Immunology, Microbiology and Parasitology, Faculty of Science and Technology, University of the Basque Country (UPV/EHU), 48940 Leioa, Spain; leire.martin@ehu.eus (L.M.-S.); andoni.ramirez@ehu.eus (A.R.-G.); 3 Department of Gastroenterology, University Hospital of Basurto, Avda Montevideo 18, 48013 Bilbao, Spain; mariadelcarmen.munozvillafranca@osakidetza.eus

**Keywords:** inflammatory bowel disease, molecular mechanisms, biomarkers, therapeutic targets, Crohn’s disease, ulcerative colitis, genetic, immunity, microbiota, environmental, antibodies

## Abstract

In inflammatory bowel diseases (IBDs), such as Crohn’s disease (CD) and ulcerative colitis (UC), the immune system relentlessly attacks intestinal cells, causing recurrent tissue damage over the lifetime of patients. The etiology of IBD is complex and multifactorial, involving environmental, microbiota, genetic, and immunological factors that alter the molecular basis of the organism. Among these, the microbiota and immune cells play pivotal roles; the microbiota generates antigens recognized by immune cells and antibodies, while autoantibodies target and attack the intestinal membrane, exacerbating inflammation and tissue damage. Given the altered molecular framework, the analysis of multiple molecular biomarkers in patients proves exceedingly valuable for diagnosing and prognosing IBD, including markers like C reactive protein and fecal calprotectin. Upon detection and classification of patients, specific treatments are administered, ranging from conventional drugs to new biological therapies, such as antibodies to neutralize inflammatory molecules like tumor necrosis factor (TNF) and integrin. This review delves into the molecular basis and targets, biomarkers, treatment options, monitoring techniques, and, ultimately, current challenges in IBD management.

## 1. Inflammatory Bowel Disease

The autoimmune disorders characterized by non-infectious chronic inflammation that mainly affects the lining of the gastrointestinal (GI) tract are classified into the group of inflammatory bowel diseases (IBDs) [1,2,3]. This condition, notorious for its chronic, progressive, and relapsing nature, significantly impacts the quality of life of the patients through their immune dysregulation and the resulting inflammatory dysfunction [4,5].

The predominant IBD disorders are Crohn’s disease (CD), ulcerative colitis (UC), indeterminate colitis (IC), and unclassified colitis (IBD-U) (other non-infectious inflammations of the bowel), all of them presenting certain clinical and histopathological similarities but affecting different regions of the GI tract. UC primarily targets the mucosa of the colon in a continuous pattern, whereas CD can affect any part of the GI tract, from mouth to anus, in a discontinuous pattern [1,6,7]. Within both CD and UC, various subtypes exist. UC subtypes are based on the affected area, such as proctitis (limited to the rectum), proctosigmoiditis (extending into the sigmoid), distal ulcerative colitis (beyond the sigmoid), or pancolitis (involving the entire colon up to the cecum). Conversely, CD is classified according to phenotype, including inflammatory, structuring, or penetrating presentations [1]. Since 2005, the Montreal classification of IBD has been utilized as a standard framework for categorizing the disease according to its clinical, molecular, and serological features [8,9,10].

No cure exists for IBD, necessitating symptomatic treatment strategies that primarily aim to reduce inflammation and promote gut healing [11,12,13]. However, the administration of these medications often entails side effects and may lead to clinical failure or loss of response, emphasizing the necessity of diligent monitoring and the exploration of more efficient treatment modalities [12,14,15,16,17,18].

The clinical presentation is wide, as patients can experience diarrhea, abdominal pain, rectal bleeding, and weight loss [11,19]. Beyond its primary impact on the GI tract, IBD may present extraintestinal manifestations (EIMs) in approximately 25% to 40% of patients; as such, this disease is classified as a systemic disease. The EIM can vary widely among patients and often includes fatigue, IBD-related arthritis, anemia, oral aphthous ulcers, pyoderma gangrenosum, fever, nephrolithiasis, osteoporosis, anterior uveitis, and erythema nodosum, among others [4,6,11,19,20]. The wide range of clinical presentations associated with IBD diseases delays diagnosis and renders diagnosis challenging; hence, understanding them is necessary for identifying novel and valuable biomarkers and therapeutic options [21,22].

**Figure 1:**
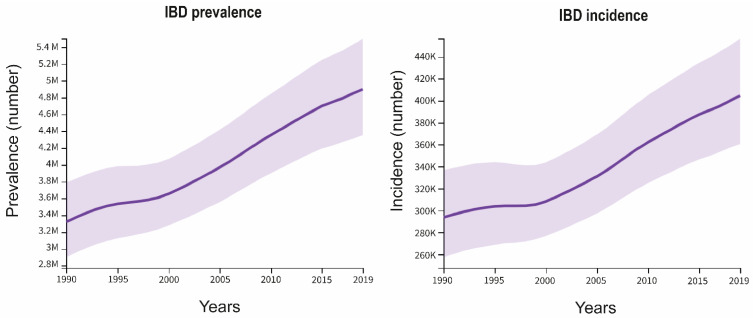
Prevalence (**left**) and incidence (**right**) trends from 1990 to 2019. Charts acquired from Global Burden of Disease (GDB).

The incidence and prevalence of IBD have seen a notable increase over the last decades since its emergence in the 20th century (Figure 1), specifically in industrialized nations, such as North American and European countries. Indeed, the highest prevalence is observed among Caucasians. Nevertheless, the global burden of IBD has increased due to its growth in newly industrialized countries, like those in Asia, Africa, and Latin America, and in immigrant populations that move to industrialized countries [3,11,19,23,24]. Based on the 2019 Global Burden of Disease (GBD) findings [25], the prevalence of IBD was estimated to affect approximately 5 million individuals, with an annual incidence of approximately 400,000 new cases reported. Furthermore, age and sex influence the incidence and prevalence of IBD. For example, disease onset occurs during childhood in 25% of cases, with its incidence continuing to rise [12,23,26,27]. In Europe, the EpiCom/Epi-IBD study reported an incidence of 15 cases/100,000 person-years [28]. Although the incidence seems to be stabilizing in the Western world, the prevalence of IBD continues to rise. This trend is expected, as IBD typically starts at a young age, has low mortality, and cur-rently lacks a curative treatment [29]. Therefore, the increasing prevalence of IBD highlights the need for a better understanding of its molecular bases to develop targeted therapies and diagnostic tools.

Moreover, this variation in incidence and prevalence across different populations suggests the involvement of both genetic and environmental factors, which can be defined as crucial areas for research into molecular targets. Indeed, although IBD etiology remains unclear due to its intricate and multifactorial nature, it is known that there is a combination of genetic and environmental factors. These factors lead to an uncontrolled activation of the intestinal immune system against commensal microbiota and other targets [30,31,32,33,34]. Regarding the genetic factors, they appear to be pivotal to the onset of IBD. Approximately more than 200 loci have been related to IBD susceptibility through genome-wide association studies (GWASs), which will be discussed later, but there is ongoing research aimed at uncovering additional genetic markers [6,30,31]. Concerning environmental factors, the observed rise in IBD prevalence, particularly in industrialized countries, suggests the important role of environmental influences in its development. Among these, smoking, alcohol, drugs, and diet are the most significant. The hypothesis suggests that all of these factors modify the GI lining and disrupt the gut microbiota, a complex ecosystem of microorganisms colonizing the GI tract. Certainly, disruptions of the gut microbiota have been proposed as prominent environmental triggers for IBD, potentially contributing to chronic inflammation [17,30,35]. The adaptive immune response of patients is also implicated in IBD, as it involves an amplified response of immune cells leading to the secretion of pro-inflammatory cytokines, which in turn triggers inflammation. Along with the adaptative immune response, the intestinal barrier and antigen-presenting cells (APCs) also play an important role in the development of this disease [6]. Indeed, it is important to note that a notable proportion of the genes associated with IBD are related to immune function, including, specifically, the host mucosal barrier function and its interaction with the microbiota. To sum up, genetic factors trigger immunological dysregulation, which, in conjunction with microbial and environmental influences, leads to the development of IBD [30,33,36,37,38].

In light of the preceding considerations, this review intends to offer a comprehensive analysis of the molecular bases, biomarkers, diagnostic methods, and therapeutic options of IBD, which may assist other researchers in understanding the disease’s background. We will conclude by outlining the remaining challenges and unmet clinical needs in this complex group of diseases.

## 2. Underpinnings of IBD: From Molecular to Environmental Factors

The intestine is a physical and biochemical barrier comprising certain crucial components: intestinal epithelial cells (IECs) tightly bound to each other, surface mucus secreted by globet cells, normal peristalsis, the microbiota, immune cells, and numerous protective factors. Moreover, in the gut mucosa, immune cells can be found in organized secondary lymphoid structures, known as gut-associated lymphoid tissue (GALT), in intestinal-tissue-draining mesenteric lymph nodes, between surface epithelial cells, and within the underlying connective tissue. In healthy individuals, these components collaborate to uphold the integrity of the barrier, preventing its disruption and the onset of diseases, such as IBD [39,40,41,42,43].

The aforementioned aspects of the intestinal barrier play a pivotal role in the pathogenesis of IBD as they significantly influence the susceptibility to local and systemic inflammation. Disruption of this barrier by environmental factors in genetically predisposed individuals may allow the translocation of commensal bacteria into the intestinal lamina propria, potentially triggering immune responses and perpetuating the inflammatory cascade [40,44].

**Figure 2:**
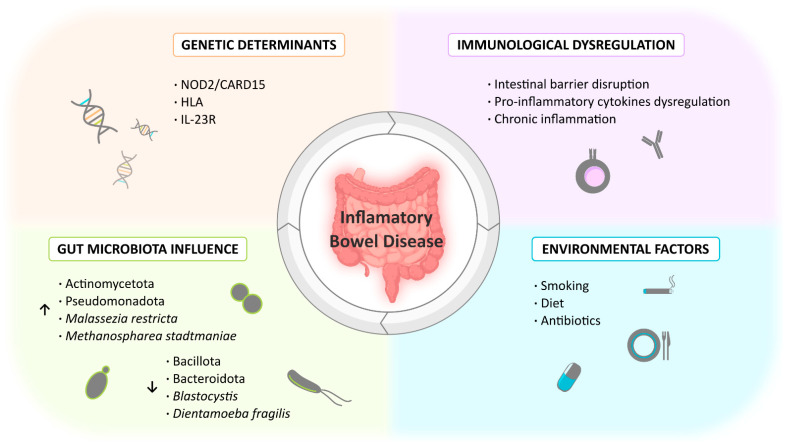
Most relevant immunological, genetic, environmental, and microbiota influences.

Hence, it is crucial to comprehend the dysregulated molecular mechanism within the intestine environment that contributes to the development of IBD. Similarly to the factors influencing its etiology, research must delve into genetic, immunological, environmental, and microbiota influences (Figure 2).

### 2.1. Genetic Determinants

The most popular technique to provide evidence of causality using genetic proxies for putative risk factors in instrumental variable analyses, GWAS, has defined more than 240 allelic risk variants linked to IBD in genes encoding proteins involved in both innate and acquired immune responses [40,45,46]. Research studies have indicated a heritable risk, which is more pronounced for CD compared to UC [47]. Indeed, the influence of genetic factors in IBD explains the higher risk among first-degree relatives of patients with IBD and the greater concordance in homozygous twins. However, most cases of IBD have a multigenic origin [47].

The first and most well-established gene with the greatest impact on IBD is caspase activating recruitment domain 15 (CARD15), also known as NOD2 (nucleotide-binding oligomerization domain-containing protein 2), which was discovered by Ogura et al. [48] within the IBD1 loci on chromosome 16q12 [38,40,49]. Nowadays, it is defined as a gene that encodes a protein in leukocytes whose function is immunological, as it is a pattern recognition receptor (PRR), within the group called NOD-like receptors, which recognize molecular patterns present in microorganisms and trigger the innate immune response. Subsequently, it activates the nuclear factor κB (NF-κB) protein, regulating the expression of tumor necrosis factor (TNF), chemokines, antimicrobial peptides, and other pro-inflammatory cytokines [50,51,52,53,54,55]. Hence, this gene maintains intestinal barrier integrity by regulating the composition of the intestinal microbiota and modulating both innate and adaptative immune responses [50]. Variants of the NOD2 gene result in unresponsiveness to pathogens, allowing them to penetrate the gut and triggering the inflammatory response. Indeed, this gene has been linked to ileal CD (with a three-fold increased risk), earlier age at diagnosis, a higher incidence of the disease, and the need for surgery [56]. This gene has also been related to sacrolitis and uveitis EIM of IBD [4].

Additionally, the IBD3 locus situated on chromosome 6p21 has been identified as a susceptibility locus for IBD. This locus encompasses genes encoding the major histocompatibility complex (MHC), including human leucocyte antigen (HLA) and class I, II, and III genes. In the latter class, non-classical proteins from the MHC, such as TNF and other immune-related proteins, are encoded, but these are not HLA genes themselves [37,49,57]. Regarding TNF, it seems to be the principal pro-inflammatory cytokine involved in IBD development, as it promotes inflammation by inducing the production of other pro-inflammatory cytokines, such as IL-1β and IL-6. TNF stimulates the uptake of epithelial antigens in the ileum. Several polymorphisms have been found in this gene, which is related to IBD, such as the TNF-α-238G/A [58], 308 G/A [58,59,60,61], -857 C/T [60,62], or 1031T/C [58,63], as well as TNF superfamily member 15 (TNF15) [64,65]. Additionally, TNF has been proposed to contribute to the onset of EIMs [66,67,68].

One of the functions of HLA genes is their role in the differentiation of self- and non-dangerous tolerable antigens from those that are extraneous, against whom a response must be initiated. The involvement of HLA variations in IBD is hypothesized to stem from an aberrant function of these genes, leading to an immune response against key bacteria of the gut microbiota and self-antigens owing to cross-reactivity. HLA genes play a more modulatory role rather than direct susceptibility to the disease, and they are more relevant in UC than in CD [56]. Certain variants of specific alleles (e.g., rs2647087 [69], rs2395185 [12], and rs2097432 [12]) have been associated with the development of immunogenicity against anti-TNF treatments, the requirement for surgery, or the severity of the disease [12,49,70]. Furthermore, specifically, HLA-DRB1 is identified as one of the primary genes associated with UC, along with HLA-DQA1 and HLA-DQB1 genes showing robust associations [38,61,70]. In contrast, for CD, polymorphisms in the HLA-G and HLAB21/Cw8 genes are more strongly associated than those observed in UC, although both UC and CD have shared risk loci and gene variants [37,38,71]. In addition, HLA genes were associated with EIMs in several studies, as well as with the anti-TNF therapy loss of response owing to antidrug antibody development [4,72]. For instance, Musculoskeletal EIMs in CD are linked to HLA-A2, HLA-DR1, and HLADQw5, whereas DRB1*0103, B27, and B58 alleles with UC [4].

Following the IBD-associated loci, the locus IBD 1 to 27 were described, although it is not a usual classification as it is preferred to define the gene and not the loci [73,74]. For example, within the IBD5 locus (5q31), genes, such as interferon regulatory factor 1 (IRF1) and solute carrier family 22 member 4 (SLC22A4, also known as Carnitine/organic cation transporter 1 (OCTN1)) and 5 (SLC22A5, also known as OCTN2), have been identified, all of which are related to these disorders [75,76,77].

Additional genes associated with IBD development and the immune response, pathogen detection, or intestinal barrier integrity include IL10 [38,78,79], IL-10 receptor (IL10R) [38,79,80], IL-23 receptor (IL23R) [38,40], IL-1 receptor alpha (IL1RA) [80], autophagy-related 16-like 1 protein (ATG16L1) [38,81], immunity-related guanosine triphosphate M (IRGM) [38,81], Protein tyrosine phosphatase non-receptor type 2 (PTPN2) [38,80], cadherin 1 (CDH1) [38], hepatocyte nuclear factor 4-alpha (HNF4-α) [38,40], and unc51-like autophagy kinase 1 (ULK1) [43], among others. It should be noted that the IL23R gene, located on chromosome 1, presents some variants (e.g., Arg381Gin) associated with a decreased risk of IBD as those alter receptor expression and function. Hence, IL-23 pro-inflammatory signals are not effectively transduced, leading to a decreased inflammatory response, disruption of a wide variety of signaling pathways, and a failure of cell functions [56,82].

Regarding IBD EIM-associated genes, IL-8 receptor alpha (IL8RA), positive regulatory domain I (PRDM1), ubiquitin-specific protease 15 (USP15), and tissue inhibitor of metalloproteinase 3 (TIMP3) genes are related to pyoderma gangrenosum, integrin subunit beta 3 (ITGB3), suppressor of cytokine signaling 5 (SOCS5), C-type lectin domain family 4 member K (CLEC4K or CD207), integrin alpha L chain (ITGAL), Prostaglandin E receptor 4 (PTGER4) with erythema nodosum, primary sclerosing cholangitis with tyrosine kinase 2 (TYK2), signal transducer and activator of transcription-3 (STAT3), Janus kinase 2 (JAK2), SOCS1, Forkhead box protein O1 (FOXO1), interferon regulatory factor 8 (IRF8), Bcl-2-like protein 11 (BCL2L11), and ubiquitin associated and SH3 domain containing A (UBASH3A) [4].

However, depending on the ethnicity, the significance of the allele presence in the patient could vary for all of the mentioned variants [40,65,81]. For example, NOD2 variants are present in European patients but not in Asiatic patients [40,81]. Furthermore, depending on the gene variant, it could be a risk or a protective factor against IBD development, and it can also determine the severity of the disorder [38,40]. Thus, gene associations differ not only between CD and UC but also among different disease presentations, such as the HLA-DQA1*05 allele, which has been associated with the development of antidrug antibodies [72], and different ethnicities. On the other hand, it should be noted that depending on the affected allele, the function will be different.

In addition to immune-related genes, certain genes encoding mitochondrial proteins have also been identified as IBD susceptibility genes. Indeed, mitochondria are increasingly recognized as integral to immune response signaling. Experimental studies have revealed a downregulation in mitochondrial genes and a correlation between the tight junctions of the IECs and mitochondrial dysfunction [83,84,85]. Acyl-CoA dehydrogenase medium chain (ACADM) [83], pyruvate dehydrogenase kinase isozyme 1 (PDK1) [83], and fission 1 (FIS1) [83] mitochondrial genes are associated with UC, SLC22A5 [85,86], laccase domain containing 1 (LACC1, also known as C13orf3 and fatty acid metabolism-immunity nexus (FAMIN)) [85,87], glutathione peroxidase 1 (GPX1) [85], and GPX3 [85,88] with CD, while aldehyde dehydrogenase 2 family member (ALDH2) [64,85] and STAT3 [85,89] are associated with IBD in general.

Interestingly, recent studies [84,85,90] have revealed that mitochondrial PARK7 (Parkinson’s disease 7) and LRRK2 (leucine-rich repeat kinase 2) genes are related to an increased risk of IBD. While these genes are primarily linked to Parkinson’s disease (PD), they are also implicated in the regulation of the immune system, mitophagy, and maintaining mitochondrial balance, making their dysregulation a risk factor for IBD and other autoimmune disorders [81,83,91]. Likewise, PARK7 deficiency is thought to elevate p53 levels, a protein that induces apoptosis in IECs. Consequently, adequate expression of PARK7 not only provides a defense mechanism against oxidative stress but also protects the mucosal barrier [83,92,93]. Various epidemiological and experimental studies have linked IBD with neurodegenerative diseases, such as PD. In addition to PARK7 and LRRK2, the genes NOD2, GAK, HLA-DRB5, and MAPT are present in both IBD and PD [90,94].

A table (Table 1) has been added to simplify the explained information. This table provides details about the gene loci, their functions, and the associated diseases.

It is interesting to note that IBD-associated genetic variants may have important evolutionary implications, potentially persisting in the genetic pool due to their dual impact. While a variant may increase the risk of IBD, it could also confer benefits for specific immune functions, such as the HLA class II DRB1*04 allele [49], which increases the risk of present UC but is protective against primary sclerosis cholangitis. In addition, the onset of this disease is multifactorial, typically requiring the interplay of multiple genetic variants, environmental factors, and immunological dysregulation [36]. Likewise, novel therapeutic options, particularly the innovation of anti-TNF treatments, enable patients to live longer and potentially pass on their genes to their offspring [96].

### 2.2. Immunological Dysregulation

The immune system is responsible for protecting the organism against pathogens (bacteria, fungi, viruses, or parasites), harmful substances from the environment, and cell changes that can become cancer cells [40,97]. Because it is a very complex and coordinated system, for its study and understanding it can be divided into two subsystems: the innate immune system and the adaptative immune system. On the one hand, the innate immune system is the first and nonspecific defense, providing the initial response to microorganisms and other external particles. It is composed of physical barriers (intestinal mucosa, skin, etc.), physiological barriers (low pH, temperature, or chemical mediators), small molecules (complement, defensins, etc.), and innate immune cells, which are comprised of myeloid-derived cells (neutrophils, monocytes, dendritic cells (DCs), and macrophages), and innate lymphoid cells, including natural killer (NK) cells [40,98,99]. On the other hand, the adaptive immune system is a specific response against the antigen, and it is composed of T and B lymphocytes [97,98,100,101,102]. Likewise, the adaptive immune system is classified into two groups: humoral immunity, which is mediated by antibodies, mainly immunoglobulin (Ig) A in the gut, secreted by B cells, and cellular immunity, which is mediated by T lymphocytes that can be functionally divided into CD4+ helper T cells (Th), CD8+ cytotoxic T cells (Tc), and regulatory T cells (Treg) [39,97,101]. Th cells can be further subdivided functionally into various subsets that are, in part, defined by the cytokines they produce. These include Th1 cells, Th2 cells, Th9 cells, Th17 cells, and T follicular helper cells (Tfh), among others [103].

Furthermore, the microbiota also plays a crucial role in the induction, training, and function of the host immune system, protecting from pathogenic microorganisms and maintaining homeostasis together. Hence, the immune system develops a tolerance to this microbiota, creating a useful symbiotic relationship [104,105,106]. In fact, microbiota could even be considered a biological barrier of the innate immune system complementing the physical and physiological barriers mentioned above. Disruption of this relationship triggers an inflammatory response that can lead to various diseases. Indeed, many studies support the idea that IBD results from a dysregulated response by the mucosal immune system to the microbiota of the intestinal lumen [40,104].

One of the main theories about the development of IBD in genetically susceptible individuals is that certain environmental factors can compromise the integrity of the intestinal barrier and intestinal microbiota, leading to the activation of inflammatory mechanisms [34,40].

Initially, epithelial disruption increases autophagy and apoptosis, while colonocyte differentiation is decreased. Consequently, surface mucus production becomes defective, and tight bounds between IECs start to increase permeability [81,107]. This permeability is also enhanced by abnormal regulation of genes (e.g., WFDC2 downregulation) and some specific gene variants (e.g., HNF4A, CDH1, or LAMB1), which also compromise tight junctions [40,106]. Likewise, pro-inflammatory cytokines released during inflammatory mechanisms (such as TNF, interferon-gamma (INF-γ), IL-1β, or IL-13) further increase barrier permeability by affecting tight junctions, IECs, and protein from membranes, while also inducing apoptosis of IECs, as observed with IFN-γ and IL-13 [40,106]. Moreover, NOD2 defective variants result in intestinal dysbiosis that reduces butyrate oxidation, leading to an epithelium deprived of energy and degrading the mucus, which exposes more of the IECs to luminal agents. Consequently, it heightens their exposure to microbial signals, further prompting the secretion of inflammatory cytokines [104,108]. Interestingly, this decreased oxidation also elevates oxygen levels, promoting a more aerobic environment that favors dysbiosis with aerobic bacteria [104].

Upon gut barrier disruption and immune system dysregulation, microorganisms can penetrate the epithelial barrier and infiltrate the intestinal lamina propria. Then, DCs are attracted to the inflammatory site by CCL20 and addressins [40]. Certainly, elevated recruitment and activation of various immune cell subsets are evident in IBD. Specifically, in the lamina propria of IBD patients, myeloid cells exhibiting an “inflammatory” phenotype, marked by heightened cytokine production, have been identified [99].

In the lamina propria, DCs and macrophages recognize pathogen-associated molecular patterns (PAMPs), such as lipopolysaccharides (LPSs) and flagellin, through their PRRs, like toll-like receptors (TLRs) or NOD-like receptors (NLRs), to phagocytize them [106,109]. Subsequently, DCs and macrophages release pro-inflammatory cytokines, such as TNF and many interleukins (IL-1β, IL-6, IL-8, IL-12, IL-18, and IL-23).

Among them, IL-1β and IL-18 are produced by the inflammasome, which is chronically activated in IBD, exacerbating the inflammation and tissue damage [110]. Moreover, IL-1β induces the release of IL-17 and IFN-γ in immune cells, such as T cells and innate lymphoid cells, which also contribute to maintaining inflammation [106,110]. Regarding IL-23, it activates STAT-4 in memory T cells, which in turn induces the production of IFN-γ, thereby increasing inflammation [111]. This is why IL23R variants, in which the cytokine receptor is not properly activated, serve as a protective factor for both CD and UC. Similarly, NK cells can be activated by cytokines, e.g., IFN-γ or IL-23, releasing inflammatory cytokines and toxic particles and inducing apoptosis [57,106]. APCs also secrete cytokines that exacerbate inflammation and stimulate the adaptive immune response [106]. Although in healthy patients intestinal DCs usually produce anti-inflammatory IL-10 to maintain homeostasis, in IBD patients, its production is decreased. This reduction is partly due to a decline in *Bifidobacterium* and *Saccharomyces cerevisiae*, which promotes its production, but it may also be a consequence of the presence of an IL-10 gene variant [40,78,106,112].

In IBDs, immune system dysregulation that originates in the innate response subsequently extends to the adaptive response. This latter reaction is directed against self-antigens, triggering chronic inflammation [40,111]. The adaptive immune response starts once an antigen is detected by DCs and presented to T helper 0 or naive (Th0) cells in GALT [106,113]. However, in individuals with IBD-associated risk HLA alleles, a residue change in the antigen-binding domain may lead to the presentation of incorrect peptides to T cells. This incorrect presentation can contribute to the activation of auto-reactive T cells [114]. Subsequently, the immune system is activated against both intestinal and non-intestinal antigens, with the latter contributing to EIMs [4].

Then, depending on the environmental cytokines and transcription factors, Th0 cells can differentiate into specific subsets. For example, IL-12 and IL-18 drive differentiation into Th1 cells, IL-4 promotes differentiation into Th2 cells, TGF-β and IL-6 induce differentiation into Th17 cells, TGF-β and IL-2 lead to differentiation into Treg cells, TGF-β and IL-4 drive differentiation into Th9 cells, and IL-21 drives differentiation into Tfh cells [40,106,115].

Specifically, in the case of CD, there is an exacerbated Th1 and Th17 response, with the latter leading to the activation of Tc cells and the excessive release of IFN-γ and IL-17 [40,106,116]. On the contrary, in UC, the induced response is mainly mediated by Th2 cells, which is characterized by IL-4, IL-5, IL-13, and IL-23 release, with IL-5 and IL-13 being particularly increased, along with the Th17 response, involving the release of IL-17 and IL-22 [40,106,116]. Similarly, the Th9 response is also related to UC, as they are responsible for regulating inflammation and anti-tumor response, secreting IL-3, IL-9, IL-10, and IL-21 [40,113].

Moreover, in healthy individuals, anaerobic commensal bacteria present in the microbiota produce short-chain fatty acids (SCFAs), which activate Treg cells and inhibit Th17 responses. However, in individuals with IBD, there is a reduction in anaerobic bacteria, leading to decreased SCFA production. Consequently, the anti-inflammatory response, mediated by IL-10 cytokine released by Treg cells, is compromised, allowing for the activation of Th17 cells. Th17 cells then produce pro-inflammatory cytokines, such as IL-17, IL-17A, IL-17F, IL-21, and IL-22. Furthermore, the reduction in commensal microbiota may disrupt the microbial balance, allowing the proliferation of pathogenic bacteria. These detrimental bacteria promote the production of serum amyloid A proteins (SAA1 and SAA2), which are associated with IBD and promote inflammatory Th17 responses [40,106].

The humoral response also participates in the pathogenesis of IBD. When an infection occurs, some of the Th0 cells undergo differentiation into Tfh cells, which subsequently participate in the activation of the B cells. These B cells further differentiate into plasma cells within GALT, where they produce immunoglobulins (Igs) [117]. In the gut of healthy individuals, these Igs regulate the penetration of microorganisms into the IECs and facilitate the presentation of antigens to the mucosal immune system, specifically secretory IgA (SIgAs) and IgM. SIgAs are predominant antibodies in the intestinal lumen, thus being considered the primary component of intestinal immunity [118]. Its abundance is due to the fact that TGF-β and retinoic acid released by DCs increase the amount of SIgA produced by triggering the isotype change of B cells and promoting their cell proliferation towards plasma cells; similarly, activated PRRs in IECs promote isotype switching [119]. One of the functions of IgAs is to maintain gut microbiota composition and eliminate pathogenic bacteria [120]. In IBD patients, increased production of IgA, particularly against pathogenic and colitogenic bacteria, has been found [94,120,121,122]. However, IgGs also play a crucial role in neonatal intestinal immunity as maternal IgGs train and develop post-natal immunity. While it was previously thought that IgGs in adults had less significance due to their potential to induce unnecessary inflammation, it is now understood that IgGs are present in the gut wall and serve an essential function in protecting the host from systemic infections [118,123,124]. Moreover, due to the compromised barrier in IBD patients, serological IgGs find it easier to infiltrate the intestinal lumen, thus increasing their presence and, consequently, inflammation [125].

In fact, some studies have revealed a variant in FCGR2A (FcγIIA-R131) responsible for encoding a receptor that binds to the Fc portion of IgGs with reduced affinity, which has been identified as protective in UC. Thus, it is proposed that IgGs play a critical pathogenic role in UC, contributing to intestinal inflammation [118,126,127]. Additionally, FCGR2A, FCGR3A, and FCGR3B expression is increased in biopsies taken from inflamed areas of UC patients compared with those in healthy patients. Hence, it is proposed that IgG binding is related to inflammation and, consequently, the development of UC [126]. Similarly, IBD patients exhibited higher levels of food-specific and non-commensal-bacteria-specific IgGs in their serum [118,125,128]. Nevertheless, due to the intensified Th2 response in UC and Th1 response in CD, the latter condition yields fewer autoantibodies but higher levels of IgG and IgA against *S. cerevisiae*, flagellin, and *E. coli*. Conversely, UC patients generate more anti-neutrophil cytoplasmic antibodies (ANCAs) and antibodies against tropomyosin 1 and 5 isoforms [120,129].

Thus, different factors induce immune system dysregulation, triggering an increased inflammatory immune response and recruiting B and T cells to the lamina propria, which in turn exacerbate the inflammatory response. Identifying the precise triggers of IBD is challenging due to its multifactorial nature, involving genetic, immune, microbial, and environmental factors. Rather than a single trigger, a combination of these elements interacts to drive the development of the condition. This complexity makes it difficult to determine whether damage to the intestinal barrier initiates an intensified inflammatory response or if this barrier damage results from heightened inflammation and changes in the microbiota. In any case, the immune system’s inability to regulate this inflammation causes its persistence and chronic spread, culminating in the characteristic IBD symptoms [29,36].

**Figure 3:**
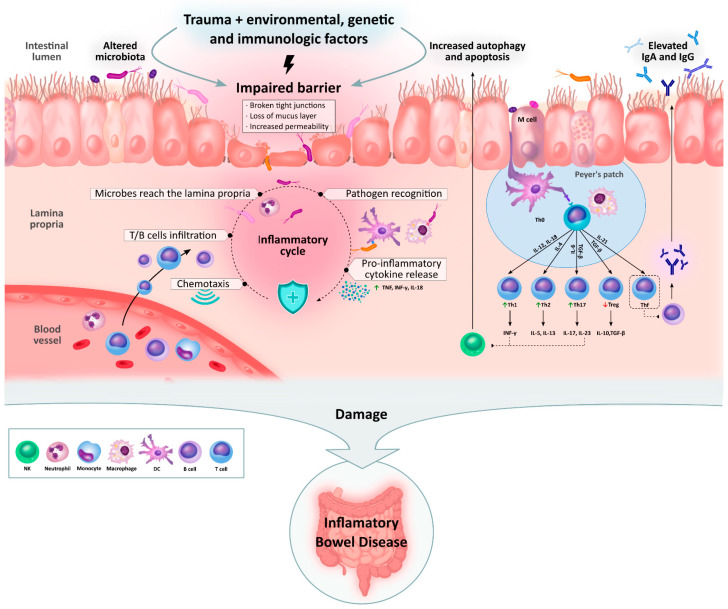
Overview of key inflammatory events initiating inflammation. Initially, environmental, genetic, and immunological factors disrupt the intestinal epithelial barrier, allowing microbiota entry into the lamina propria and triggering an inflammatory cascade. Dendritic cells (DCs) and macrophages recognize pathogens, presenting antigens to T and B cells, resulting in cytokine release. In Peyer’s patch, a gut-associated lymphoid tissue (GALT), DCs stimulate Th0 cell differentiation into specific T cell subtypes based on environmental cytokines. B cells produce elevated levels of immunoglobulins (Igs) A and G in the intestinal lumen, while natural killer (NK) cells promote autophagy and apoptosis.

The key aspects of immunological dysregulation during IBD are represented in the following figure (Figure 3).

### 2.3. Gut Microbiota Influence

The human microbiota is composed of approximately 10^13^ to 10^14^ microorganisms [130,131,132,133], surpassing the total number of human cells. Thus, it is predictable that the microbiota can be described as a pivotal element in the onset of IBD. This microbiota includes viruses, archaea, bacteria, fungi, and protozoa [134,135,136]. The majority of microbiota studies are focused on bacteria, with the most prevalent phyla being Bacillota (previously known as Firmicutes) (*Enterococcus*, *Lactobacillus*, *Streptococcus*, *Ruminococcus*, *Clostridium*), Bacteroidetes (*Prevotella*, *Porphyromonas*), Actinobacteria (*Bifidobacteria*), and Proteobacteria (*Escherichia coli*) [30,137,138]. Consequently, a significant portion of the existing literature pertains to bacterial aspects.

IECs produce the mucosal barrier to protect the host from microorganisms, reducing the gut barrier’s permeability and avoiding the contact of microorganisms with immune cells [139,140,141]. Nevertheless, microorganisms of a healthy microbiota have a crucial role in the normal immune response, modulating it and maintaining its homeostasis along with the intestinal epithelial barrier [134,139,142]. For example, microorganisms of the GI tract produce important molecules during their metabolism that support host immune functions, aiding in the cross-talking between IECs and immune cells, but they also avoid opportunistic infections by niche competition [135,143]. Likewise, they modulate the production of certain immune cells, antibodies, and antimicrobial peptides [140]. Besides the immunological support, they help host intestinal tissue development, nutrient absorption, metabolic actions, and neurological functions [134,143,144].

For instance, SCFAs, mainly propionate, acetate, and butyrate, are important metabolites produced by the healthy microbiota [131,139,141]. Certain bacteria fermentate complex carbohydrates and create those SCFAs, e.g., *Bifidobacteria* species, *Faecalibacterium prausnitzii*, *Roseburia intestinalis*, and *Anaerostipes butyraticus* [139]. This metabolite holds significant relevance due to several characteristics: (1) its abundance; (2) its ability to activate, regulate, and differentiate various immune cells, including Treg cells, which elicit an anti-inflammatory response; (3) its direct anti-inflammatory impact, controlling the production of pro-inflammatory cytokines like IL-6, IL-12, and TNF, while promoting the generation of the anti-inflammatory IL-10; (4) its role in protecting against colonization by harmful microorganisms, achieved through actions like pH reduction; (5) its provision of a carbon source to colonocytes; and (6) its contribution to maintaining the intestinal barrier integrity by activating G-protein-coupled receptors (GPCRs) and regulating cellular turnover [131,139,140,145]. Specifically, *Bifidobacterium* produces acetate at high levels, which binds to GPRCs and reduces NOD and LRR- and pyrin-domain-containing protein 3 (NLRP3) inflammasome activation with its secretion of IL-18, a crucial pro-inflammatory cytokine in colon inflammation, as well as acetate, which attenuates NF-kB expression [139,145]. Interestingly, SCFAs also control gene expression, as they inhibit histone deacetylases (HDACs), which eliminates acetyls from DNA, inactivating its gene expression. Conversely, histone acetyltransferase (HAT) activity, which has the opposite function, is stimulated, and immune tolerance that produces anti-inflammatory cell phenotypes is generated [139,140,145].

Commensal bacterial cells also avoid opportunistic or pathogenic bacteria colonization by competing for nutrients [30,138]. For instance, residual oxygen consumption by commensal microbiota is also an essential part of the host defense. The healthy microbiota is mainly composed of obligate anaerobes but also facultative anaerobes, which consume that free oxygen, generating an environment without oxygen in which harmful metabolites cannot be produced by certain pathogens, such as the virulence factors of *Shigella flexneri* [134,141].

The production of bacteriocins, lactic acid, and hydrogen peroxide is another factor that directly impacts pathogens and opportunistic microorganisms, along with SCFAs [30,138,146]. Bacteriocins, which are antimicrobial peptides, have the ability to inhibit the growth of pathogenic bacteria or even cause their death. Notably, bacteriocin producers, predominantly lactic acid bacteria (LAB) though also found in *Bacillus subtilis* and *Wissella confuse*, exhibit resistance to these antimicrobial agents [138,147,148]. Hydrogen peroxide acts by damaging proteins, lipids, and DNA of pathogenic bacteria, while LAB possesses mechanisms to avoid this oxidative stress [149,150].

Patients with IBD are associated with reductions in the total number, diversity, and richness of microbial species in their gut microbiota compared to a healthy and balanced microbiota, a phenomenon known as lower α-diversity [151,152,153,154]. For instance, Bacillota (especially *Clostridium* clusters IV and XIV) and Bacteroidota, obligate anaerobes that produce SCFAs as a part of the commensal microbiota, are found in lower proportions in patients with IBD, while Pseudomonadota (previously known as Proteobacteria) and Actinomycetota (Actinobacteria) are present at higher levels [151,153,155]. In fact, *Bifidobacterium longum*, of the class Actinomycetota, has been associated with a protective factor against UC because it reduces the production of TNF [156].

Clostridia is the class that has been described to suffer the most important modifications, decreasing the *Roseburia* and *Faecalibacterium* genus (especially *Faecalibacterium prausnitzii*, the main butyrate producer and anti-inflammatory commensal) [40]. Indeed, *Faecalibacterium* has been related to the response to certain biological drugs, such as ustekinumab. This is attributed to the increased microbiota diversity observed in patients in remission 6 weeks post-treatment compared to those with active CD [152,157].

These alterations are also reflected in the fecal microbiota. In samples from patients with CD, a reduction in the genera *Bacteroides*, *Eubacterium*, *Faecalibacterium*, and *Ruminococcus* has been observed [158,159,160]. Conversely, pathogenic microbiota families, such as *Entereobacteriaceae*, *Fusobacteriaceae*, *Veillonellaceae*, and *Pasteurellacaea*, have been described to increase [151,153,161]. Specifically, *Mycobacterium avium* subspecies *paratuberculosis* and adherent-invasive *E. coli* increments are related to CD, while *Clostridium difficile* increase is associated with relapse and remission states of both CD and UC [40]. Additionally, the molecular marker (csep1–6bpi) of *Campylobacter concisus* has been associated with active CD [152,162]. Furthermore, fecal samples from IBD patients have shown an increase in sulfate-reducing bacteria, leading to higher hydrogen sulfate production and consequent inflammatory processes in the intestinal mucosa [151,163].

In addition to bacteria, it is essential to recognize that the microbiota also encompasses viruses, such as phages, which act as bacterial parasites. Indeed, the gut virome contains 10^15^ bacteriophages, with Caudovirales, Microviridae, CrAssphages, and Gubaphages ranking among the most abundant. These phages play a pivotal role in shaping the composition of the bacterial communities and maintaining their stability and composition [164,165,166,167].

Viruses also bolster host immunity. For instance, research indicates their ability to modulate cytokine production or avoid infections; for example, they bind to mucus glycoproteins, thereby contributing to the establishment of an antimicrobial barrier that hinders bacterial infiltration into the lumen [165,168]. However, phages can confer specific characteristics to pathogenic bacteria, heightening their resistance to acidic pH, exemplified by *E. coli* infected with bacteriophage ϕ24 [165]. Various factors produced by phages may adversely impact the immune response, influencing non-commensal adhesion, colonization, and production of toxins. As an example, the ankyrin protein (ANKp), encoded within viruses, facilitates *E*. *coli* adherence to IECs, reducing their innate defense against this pathogen; likewise, adenosine-diphosphate-ribosyltransferases (ADPRTs), enzymes also encoded in phages, enhance the adherence of *Clostridium difficile* on the host mucosa [165,169,170,171]. Regarding IBD, eukaryotic viruses, such as Epstein–Barr virus (EBV) and cytomegalovirus (CMV), have been suggested as markers, but studies exploring this connection reported inconclusive findings [142].

On the other hand, although fungal microorganisms may be fewer in number compared to bacteria, their larger size renders the mycobiome equally essential to the bacteriome [172]. Similarly to bacteria, commensal fungi of the GI tract may influence host immunity, enhancing the host’s immune development, as well as helping with other functions, like metabolisms [172,173]. Nonetheless, there is a pressing need for deeper exploration of commensal fungi and their beneficial contributions to the host, as fungi are predominantly investigated as pathogens and bacteriomes in supporting the host. The main fungal phyla found within the GI tract are Ascomycota (constituting 70%), Basidiomycota (30%), and, occasionally, Zygomicota, with prevalent genera including *Candida*, *Saccharomyces*, *Malassezia*, *Penicillium*, *Trichosporon*, and *Cladosporium* [132,137,172]. However, in certain diseases, this proportion is altered. Specifically in IBD, the Basidiomycota/Ascomicota ratio is increased, as well as the *Candida albicans* proportion; however, the *S. cerevisiae* proportion is decreased [174,175]. Likewise, higher abundance and diversity of fungal microorganisms are closely related to IBD outbreaks, but researchers cannot identify a specific species related to IBD onset, as the results show discrepancies [176]. Despite the latter point, *Malasssezia restricta* is a fungus associated with CD, which enhances inflammatory responses via CARD9 signaling [143,173]. Moreover, anti-*S. cerevisiae* mannan antibodies (ASCAs), which are produced not only against *S. cerevisiae* but also against *C. albicans*, are usually employed as serological biomarkers for IBD [176].

Similarly to fungi, protozoa have been studied as parasites; even so, some also positively influence human health, such as *Blastocystis*, *Entamoeba*, *Dientamoeba*, and *Enteromonas* species [136]. For instance, *Blastocystis*, the most abundant protozoa in humans, has recently been found to be common in both healthy and unhealthy individuals, although it has been also related to certain disorders, such as irritable bowel syndrome (IBS). Additionally, its presence is closely associated with increased microbial diversity and richness, features defined as positive for health, suggesting a potential role for *Blastocystis* in preventing the colonization of pathogenic bacteria [136,177]. Indeed, some studies found its prevalence is lower in IBD and IBS patients than in healthy individuals [136]. Lastly, another species that is known to be reduced when IBD is active is *Dientamoeba fragilis* [136,178,179]. However, additional research is still necessary to clarify protozoa functions within the human microbiota.

Finally, archaea are also part of the human gut microbiota. The predominant archaea are methanogens, particularly *Methanobrevibacter smithii* and *Methanospharea stadmanae* [133,180,181]. These microorganisms establish a positive relationship with gut commensal bacteria by consuming the excess hydrogen released through anaerobic fermentation; consequently, anaerobic bacteria increase their ATP synthesis as well as their growth in the gut [182]. However, they have also been linked to IBD, although the results are inconclusive [182]. For example, high levels of *M. stadmanae* in IBD patients induce TNF and activate DCs, while *M. smithii* is related to its remission [133,180]. Another component of the microbiota identified recently is haloarchaea [182], but further research is also needed to elucidate the role of this microbiota in the human gut.

The healthy microbiota that supports many host functions can be influenced by internal and external elements, but, due to its high resilience, it can recover its composition. These elements include the immune status of the host as an endogenous factor, and, as later explained, environmental factors, such as diet, infections, or medication intake, as exogenous ones [139,143,183]. However, when microbial dysbiosis occurs in association with disruption of the mucus layer, dysregulation of epithelial tight junctions, defects in the number and function of Paneth cells, and increased intestinal permeability, it results in increased bacterial exposure [39]. Hence, the balance of the commensal microbiota is disrupted, the host lacks crucial commensal bacteria, and, simultaneously, it creates an opportunity for potentially harmful microorganisms to inhabit the space vacated by healthy ones. These microorganisms are not only exceptionally resilient, but they can cross the epithelium, eliciting an immune response, causing intestinal inflammation, and promoting further colonization of the intestine by pathogens [39,40,139,184]. Moreover, detrimental microorganisms will produce different metabolites and functions in the host, inducing an aberrant immune response and disrupting the gut barrier. This aberrant immune response generates inflammation and oxidative stress, characteristic factors seen in IBD [33,138,139]. Likewise, because the intestinal barrier is permeable, microbial molecules may extend to other organs, generating systemic inflammatory responses and EIMs [4]. Nevertheless, it is not clear whether these microbial alterations are primary drivers of IBD or secondary to the underlying intestinal inflammation observed with IBD [39,185]. Indeed, studies in rodents suggest conflicting findings; some indicate that alterations in the microbiota precede the onset of the condition, while others propose that specific changes in other factors may initiate the disease, subsequently leading to alterations in the microbiota [186]. Therefore, further research is needed to clarify this question and clarify if the microbiota is the cause or consequence of the disease.

To sum up, the microbiota is related to IBD onset, while it also aids in the differentiation between subtypes of IBD and treatment responsiveness based on the predominant microorganisms, and predicting the risk of developing more severe disease phenotypes is among the many potential applications of these findings [33,187]. Nonetheless, the usefulness of the microbiota in clinical practice is still a long way off, as certain challenges need to be overcome. For instance, the first essential requirement is the classification of a “beneficial” and “detrimental” gut microbiota to study them along with the generated metabolites and consider the metabolic variability between strains. Likewise, no standardized protocol for processing and post-processing samples has been defined, which would definitely help in the unification and normalization of results [187].

### 2.4. Environmental Factors

The environment is acknowledged for its role in inducing epigenetic changes, which involve modifying gene expression without directly altering the DNA sequence [188]. Indeed, the influence of the environment on the onset of IBD is evident, as industrialized regions exhibit higher prevalence and incidence rates of the disease. Moreover, as areas undergo industrialization, both indices tend to rise [2,3,11].

Among the environmental factors that have been studied to understand the etiology of IBD, smoking has been extensively studied and described as a risk factor for CD and, surprisingly, a protective factor for UC [106,189,190]. In addition, in CD patients, smoking is linked to joint and skin EIMs [4]. Smoking generates contaminants that breach the GI tract, such as particulate matter, reactive oxygen species producing chemicals, and free radicals [191,192]. They promote mutations and alter immune system response, mucus production, autophagy, and gut microbiota diversity [106,193,194]. For example, they have been associated with an increase in *Clostridium* abundance and a reduction in the bacteria genus previously named *Firmicutes* (nowadays included in the unclassified Bacillota group) [106,194]. Additionally, an association has been observed between smoking and genetic deficiencies in NOD2 and IL-10. Regarding the protective role of smoking in UC, it is suggested that nicotine binds to its nicotinic receptors, inducing an anti-inflammatory response, while carbon monoxide (CO) inhibits DC antigen presentation and T cell proliferation and reduces pro-inflammatory cytokines [191,192]. Consequently, given the varying expression of receptors along the intestine and the differences in affected areas of the GI tract between CD and UC, certain receptors could be more abundant in the colon, thereby acting as a protective factor [191,192]. However, further research is needed to fully understand the relationship between smoking and IBD.

Another important factor to consider is the diet [46,106,190]. Diet can compromise the intestinal barrier and trigger pro-inflammatory effects, particularly in Western diets, which contain high levels of animal fat, sugar, refined carbohydrates, emulsifiers, and chemical additives but low fiber and vitamin content along with low fish, fruit, and vegetable intake [38,46,190,195]. While certain foods, like sucrose or nutriment with omega-6 fatty acids, can exacerbate IBD, others, such as fruits and products with D and B9 vitamins or omega-3 fatty acids, may offer protective effects against this condition [46,193]. It is interesting to note that the diet effect is co-founded by the microbiota, as diet determines the composition and function of the intestinal microbiota, which in turn is relevant to overall health [196]. Indeed, diet is the main environmental factor that modifies the microbiota [197]. Therefore, the diet effect on IBD is mediated through both the direct impact of nutrients and the indirect impact of microbiota changes.

Moreover, some specific dietary items have been associated with either UC or CD but not both. For example, vegetables and green tea are protective against UC, and fiber intake has protective effects against CD, while soft drinks are identified as a risk factor for UC, and lactose maldigestion is related to CD [46,193]. Furthermore, common food production practices involve the use of additives, pesticides, heavy metals, and other contaminants, contributing to the onset and exacerbation of IBD by altering the gut microbiota [46,106,191]. Indeed, diet plays such a crucial role that specific dietary interventions are used therapeutically. Examples include exclusive enteral nutrition (EEN) [198,199,200], an anti-inflammatory diet (IBD-AID) [30,201], an autoimmune protocol diet (AIP) [202,203], a CD exclusion diet (CDED) [204,205,206] or UC exclusion diet (UCED) [207,208], a CD-TREAT diet [209,210], a specific carbohydrate diet (SCD) [30,208,210], a semi-vegetarian diet (SVD) [210,211], and a Mediterranean diet (MD) [17,30,208], which have been proposed as beneficial dietary strategies.

It is essential to distinguish the restorative and anabolic functions of diet from pharmacological therapeutic effects. On the one hand, the impact of a beneficial diet affects the inflammatory state, helping to lower it and thus heal the damaged GI tissue, but also through beneficial changes in the microbiota that will protect the patient. In other words, it has a preventive or prophylactic effect. On the other hand, pharmacological treatments are essential as they act on molecular targets that are developing the pathogenesis of IBD [212].

Antibiotic exposure is another crucial factor in the investigation of IBD [35,106,191]. This factor is particularly meaningful as both developing and developed nations increasingly rely on antibiotics in healthcare, often overusing them, and in livestock, amplifying its impact on these specific nations [35]. Specifically, this association is stronger for CD, when employing metronidazole or fluoroquinolones, and when the use of antibiotics is during childhood [33,190]. Antibiotic exposure’s significance lies in the observed decrease in microbiota diversity and richness following antibiotic use, although the majority of healthy microbiotas can be restored after a certain period without antibiotic exposure [33,35,106].

Finally, there are many other factors associated with IBD risk, such as exposure to viruses [191], psychological stress or stressful events [189,191], contaminants [35,38,191], chemicals [191], medications, like nonsteroidal anti-inflammatory drugs (NSAIDs) and oral contraceptives [38,190,193], alcohol [189], urban living [35,190,193], the poliomyelitis vaccine [190,193] and the H1N1 vaccine [190], certain surgeries, like appendectomy or tonsillectomy, especially for CD [189,190,193], vitamin D deficiency [38,193], cesarean birth for CD [193], non-*Helicobacter pylori*-like enterohepatic *Helicobacter* species [193], sedentary lifestyle in UC [189], sleep for more than 8 h [189], neuroticism [189], allergy-associated conditions [189], and hypoxia [35]. Similarly, more protective factors have been described, such as, for instance, physical activity, particularly for CD, and appendectomy for UC, even though it is defined as a risk factor for CD [189,190,193]. Other protective factors are rural living [152], breastfeeding [35,190,193], home and bed-sharing [189,190,193], high levels of folate [193] and vitamin D [193], contacts with farm animals and pets during childhood [189,190,193], and *H. pylori* infection [190,193]. Although body fat is always a risk factor, interestingly, body mass index (BMI) is a risk factor for CD but a protective factor against UC [46]. However, it should be noted that the impact of IBD onset varies among different ethnicities [193].

## 3. Biological Markers for IBD Diagnosis and Prognosis

Biological markers or biomarkers were defined as “a characteristic that is objectively measured and evaluated as an indicator of normal biological processes, pathogenic processes, or pharmacologic responses to a therapeutic intervention” by the Biomarkers Definitions Working of the National Institutes of Health. In other words, a biomarker is a molecule, parameter, or specific element that can be measured in organisms. Additionally, they are altered in response to specific events within the organism, giving information about individual health; for example, they provide information about treatment efficacy and disease subtypes, stages, and prognosis [213]. Biomarkers can be measured in blood (serum or plasma), tissue, feces, or fluids like sweat, tears, and cerebrospinal fluid [214,215,216].

Regarding IBD biomarkers, their use is still unclear, as no biomarker can provide a precise prognosis about the disease course or responsiveness to treatment. This is largely due to its intricate molecular bases and multifactorial triggers [187,217]. However, certain biomarkers are being used in clinical routines, such as C reactive protein (CRP), erythrocyte sedimentation rate (ESR), and fecal biomarkers, like calprotectin, even if each of them presents several drawbacks, e.g., suboptimal accuracy for fecal markers, long half-life and the influence of multiple factors for ESR, or the elevated heterogeneity in the generation of CRP [217]. Fortunately, many biomarkers are under research that could be incorporated into clinical practice as studies clarify their use. In this section, the most studied biomarkers are discussed (Table 2).

### 3.1. Genetic and Epigenetic Biomarkers

The relationship between specific genes and the etiology of IBD has been extensively explored in recent years, capitalizing on valuable insights gleaned from genome studies. As explained above, the association of the NOD2 gene with CD has been well-documented, emerging as a pivotal predictive factor and linked to heightened disease complications [232]. Furthermore, individuals exhibiting genetic variability in the PRDM1 and NDP52 genes have shown susceptibility to CD [232]. The genes KIF9-AS1, LINC01272, and DIO3OS have demonstrated utility in distinguishing and detecting various types of IBDs [218]. Additionally, genes like DQ786243, CDKN2B-AS1 (ANRIL), and IFNG-AS, among numerous others, have been implicated in IBDs [219], warranting further investigation to enhance our understanding and management of these conditions.

In addition to genetic markers, Microribonucleic acid or miRNAs, a group of small, single-stranded RNA molecules typically about 22 nucleotides in length, can also be candidates to be used as markers for IBD [219,220,228]. Generally, their sensitivity value is 0.8, and their specificity value is 0.84 [217]. They circulate with remarkable stability and they are conserved in blood, but they can also be found in urine, feces, and saliva, as well as in biopsies of intestinal tissues [220,232,244]. Their expression is linked to various pathologies, including IBD [220,228,244]. Among the most relevant miRNAs found to be overexpressed in IBD in various studies are miR-21, miR-223, and miR-155, implicating them in the etiology and development of the disease [220,221,222]. Additionally, overexpression of miR-375 has been studied in patients with UC compared to those with CD and patients without IBDs [223,224], suggesting its potential use as a biomarker to differentiate between different types of IBDs.

### 3.2. Blood Biomarkers

pANCA and ASCA: pANCA constitutes a group of autoantibodies that target components within the neutrophil cytoplasm [228,245], and ASCA comprise antibodies primarily directed against the mannan of the cell wall of *S. cerevisiae* yeast [232]. Several studies have observed pANCA positivity in 60–70% of sera from UC patients, compared to lower percentages in sera from CD patients (15%) [220,225]. Conversely, ASCA positivity in CD patient sera ranges from 50 to 60%, while in UC patient sera and healthy subject sera, positivity to these antibodies is much lower (5–14% and <5%, respectively) [220,226,227]. It was determined that the pANCA sensitivity value is 0.33 and the specificity value is 0.97 for differentiating between IBD and non-IBD, while the ASCA diagnostic performance has sensitivity and specificity values of 0.4 and 0.92, respectively [217].

Several studies have shown that utilizing both antibodies together enhances and complements their diagnostic power [220,226,228,232,245,246]. Patients positive for pANCA and negative for ASCA (ASCA−/pANCA+) could be diagnosed with UC with a sensitivity of 78% and a specificity of 67%, whereas those negative for pANCA and positive for ASCA (ASCA+/pANCA−) could be diagnosed with CD with a sensitivity of 67% and a specificity of 78% [220,247]. However, other studies suggest varying specificity and sensitivity values [246,248,249]. Furthermore, the combination of these antibodies has demonstrated value in diagnosing patients with indeterminate colitis among the different IBD subtypes, CD or UC [226,246]. Elevated pANCA levels have been associated with more severe UC phenotypes, as well as treatment resistance, which could aid in identifying patients requiring early surgical intervention or immunosuppressive treatment [232,250]. Conversely, elevated ASCA levels have been linked to an earlier onset of the disease and a more fibrostenotic pattern [228,251].

CRP is a pentameric protein synthesized by hepatocytes stimulated by the release of pro-inflammatory cytokines, such as IL-1, IL-6, IL-1β, and tumor necrosis factor (TNF) [220,226,228]. During acute inflammation, infection, or trauma phases, CRP levels considerably increase, while under normal conditions they remain very low (<1 mg/L), making them widely used as a serum biomarker for inflammation [228]. In patients with IBD, elevated CRP levels have been associated with more severe histological phenotypes of the disease [220] and a higher likelihood of requiring colectomy [226], aiding in distinguishing between active and inactive IBD [232]. In contrast, decreasing levels have been linked to disease remission [228] as well as a favorable response to treatment with biosimilars, such as infliximab [220]. It is worth noting that increased levels of CRP are found in various inflammation and infection processes, so it is not specific to IBD; thus, its use as a biomarker needs to be complemented with other, more specific diagnostic tests.

Regarding pro-inflammatory cytokines, several of them are involved in the etiology of IBDs, among which TNF plays a pivotal role. This molecule is primarily expressed by active lymphocytes and macrophages [67], and it is linked to various processes, such as immune stimulation and resistance to tumors and infections, by activating neutrophils, macrophages, and other components of the innate immune system [220]. High levels of TNF have been observed in serum, feces, and mucosa samples from patients with IBDs [62]. Additionally, it has been associated with inflammation present in the mucosa of UC patients [220]. The use of TNF-neutralizing therapies, such as biosimilars, has proven to be a promising strategy in the management and control of these conditions.

In addition to TNF, numerous other pro-inflammatory cytokines released by monocytes, macrophages, neutrophils, DCs, and B cells are implicated in IBDs, such as IL-12 [229], IL-23 [230], IL-1β [215], IL-6, and IL-17, among others [220], some of which are being used as therapeutic targets to monitor IBD [230]. Indeed, one of the effects of TNF is to increase the production of these pro-inflammatory cytokines during IEC damage, which bind to their receptor on cells to activate both innate and adaptative immune responses, stimulating cells like T cells and NK cells [82,229].

Cytokine oncostatin M (OSM): OSM is a compact bundle of four helices crafted from a single polypeptide belonging to the IL-6 family [252,253]. It is produced by various cell types, predominantly hematopoietic cells and stromal cells [252,254], and it has been associated with the suppression of growth in several tumor cell lines. Elevated levels of this cytokine and its receptor (OSMR) have been observed in patients and first-degree relatives of patients with IBD compared to healthy individuals and their relatives [228,236], correlating with higher degrees of inflammation and disease gravity. Additionally, its relationship with the therapeutic response to anti-TNF biosimilars has been reported, linking it to a poorer treatment response [223,252,254]. However, it is still unclear whether this association is solely related to the levels present in the mucosa or those present in the serum, necessitating confirmatory studies [236,252,254].

Anti-outer membrane protein C (anti-OmpC): anti-OmpC is an antibody directed against porin C, a transport protein on the outer membrane of *E. coli* [231,232]. The presence of IgA antibodies against these antigens has been predominantly determined in the sera of CD patients, with a prevalence of 55%, whereas in cases of UC patients, non-IBD colitis, and healthy individuals, the prevalence was much lower. This suggests that the presence of these antibodies could be useful in differentiating CD patients from other types of aforementioned diseases [220,228,231]. Furthermore, the presence of anti-OmpC antibodies has been associated with a higher likelihood of having internal perforating CD, and thus requiring surgery [228,246].

Pancreatic antibodies (PABs): PABs target the exocrine tissue of the pancreas. The main antigens recognized by these antibodies include glycoprotein 2 (GP2), CUB, and similar domains to zona pellucida 1 (CUZD1) [228,255,256]. PABs can be useful in distinguishing between patients with CD and UC, as they are detected in the sera of CD patients at a rate of 30%, whereas their prevalence in UC patients and the sera of healthy individuals is very low [231,232]. This difference arises because it is suggested that the inflammation caused by the release of anti-GP2 antibodies is primarily located in the ileum rather than the colon due to the abundance of GP2 in the M cells of the small intestine but scarce presence in the colon [228,257]. Moreover, the high specificity (96%) of anti-MZGP2 antibodies, an isoform of GP2, has been demonstrated for CD, associating it with early disease onset and more severe phenotypes [228,232,258].

Regarding CUZD1, the presence of anti-CUZD1 antibodies in the sera from CD patients is much higher than in UC patients, and its positivity is associated with ileocolonic and perianal lesions [228,259]. Despite the aforementioned findings, their use in diagnosing and distinguishing IBD must be cautious and complemented with other diagnostic techniques.

Anti-carbohydrate antibodies: This group of antibodies consists of IgG anti-laminaribioside carbohydrate antibody (ALCA), IgA anti-chitobioside carbohydrate antibody (ACCA), and IgG antimannobioside carbohydrate antibody (AMCA), along with previously mentioned ASCA [228,232,260]. They target carbohydrate epitopes present in the cell walls of certain bacteria and fungi. Studies have shown higher levels of these antibodies in patients with CD compared to patients with UC or healthy individuals [231,233,234,235]. However, there is uncertainty regarding their diagnostic utility in differentiating between IBDs when used in conjunction with ASCA [228,231,261].

Antibodies anti-membrane antigens: Recent studies have underscored the pivotal role of reactive antibodies generated against membrane antigens in the early detection of autoimmune diseases, such as IBD, as well as their significance in monitoring, managing these conditions, and influencing treatment outcomes. While further research in this specific area is warranted, promising findings have emerged. In this context, increased reactivity of sera from patients with autoimmune disease, including UC, to kidney and spleen membrane antigens has recently been reported using cell membrane microarrays of different origins [98].

Leucine-rich α2 glycoprotein (LRG) is a 50 kDa protein consisting of eight leucine-rich domains that is produced in cells, e.g., neutrophils and macrophages, in response to pro-inflammatory cytokines, such as TNF, IL-1β, and IL-6, and cytokine-stimulated neutrophils. Specifically, in IBD patients, damaged IECs also release this protein into the serum, being a great biomarker reflecting intestinal inflammation more than CRP. Moreover, it seems to be correlated with fecal calprotectin (FC). Nonetheless, it is also increased in colorectal cancer and gastric cancer, where the GI tract is also inflamed [214,223].

Other serological biomarkers: There have been numerous others biomarkers studied, including nitric oxide (NO) [220], Th17 signature cytokine IL-17A (IL-17A) [254], IL-7 receptor (IL-7R) [254], Anti-Integrin αvβ6 antibody (αvβ6 antibody) [223], Anti-granulocyte macrophage colony-stimulating factor (antiGM-CSF) [228], Anti-I2 antibody [228], Alpha-1 antitrypsin (AAT) [232], granulocyte colony-stimulating factor (G-CSF) [232], Suppression of tumorigenicity 2 (ST2) [220], and TNF alpha-induced protein 6 (TNFAIP6) [220], among many others. These markers have been found to be elevated in the serum of IBD patients, and their investigation could be valuable to better understand and manage this disease.

Additionally, beyond molecular-based biomarkers, parameters, such as ESR level [223,237] and total white blood cell (WBC) [237], eosinophil (EOS) [237], and platelet (PLT) counts [237], are being used. Among them, ESR is the most-used test in clinical practice. It has been used to detect and monitor inflammatory activity since the 1920s. However, CRP is more employed because this protein changes quicker than the ESR parameter. Moreover, it is not specific as it can be caused not only by IBD but also other autoimmune disorders, tumors, and infections. Despite its lack of specificity, this assay is low-cost, reproducible, and combined with other tests can be useful [223,262,263].

### 3.3. Fecal Biomarkers

Fecal calprotectin: Fecal alprotectin is a heterodimeric protein belonging to the S100 family, which plays a crucial role in the mechanisms underlying both acute and chronic inflammation [220,226]. It is primarily secreted by neutrophils and exhibits antimicrobial activity by competing for zinc, a nutrient utilized by bacteria, thereby limiting their growth [264]. Elevated levels of calprotectin have been found in samples of feces, serum, and other fluids from patients with IBD compared to those obtained from healthy individuals, with the most substantial differences observed in fecal samples. Consequently, fecal calprotectin has been widely utilized as a biomarker for inflammatory states (sensitivity 0.88 and specificity 0.8) [217,220,226]. Additionally, calprotectin found in fecal samples can provide a measure of specific intestinal inflammation, whereas that obtained from other fluids offers a more general measure of body inflammation [223]. Despite being a stable biomarker, the presence of elevated calprotectin levels in other pathological conditions renders it nonspecific, thereby precluding its use solely as a biomarker for diagnosing IBD.

Fecal lactoferrin: It is a multifunctional iron-binding glycoprotein found in various exocrine secretions, such as saliva, sweat, and mucosal secretions, among others [158,220]. It has a key role in immunity, being released in large quantities by neutrophils at sites of inflammation during infections caused by bacteria, viruses, fungi, yeasts, and other pathogens [220,238,239]. Elevated levels of lactoferrin have been observed in patients with IBD compared to healthy individuals, indicating a correlation between lactoferrin levels and the degree of inflammation observed through mucosal endoscopies (sensitivity 0.82 and specificity 0.95) [158,217,220,238,239]. This correlation suggests a potential utility in assessing inflammation levels in these patients, leading to superior disease management strategies.

Calgranulin C (also known as S100A12): It is a pro-inflammatory protein predominantly secreted by neutrophils, belonging to the S100 protein family [265]. This protein exhibits antimicrobial properties attributed to its ability to chelate metals, which are essential nutrients for many microorganisms. By sequestering these metals, calgranulin C restricts their availability to pathogenic microorganisms, thereby impeding their growth [238,240]. Elevated levels of this pro-inflammatory protein have been consistently observed in the serum, mucous membranes, and fecal samples of patients with IBD compared to healthy individuals [158,238,240]. Notably, calgranulin C demonstrates uniform distribution within fecal samples and remarkable stability, persisting for up to 7 days at room temperature and withstanding degradation by fecal bacteria [265,266]. Moreover, a significant correlation has been established between calgranulin levels and the severity of both the disease and mucosal inflammation in IBD patients. These distinctive characteristics position calgranulin C as a promising candidate for an IBD biomarker; however, its clinical utility remains, as S100A12 has also been implicated in other inflammatory conditions [158,220,238].

Myeloperoxidase (MPO): MPO is a peroxidase present in many cells throughout the body, predominantly in neutrophils and immature monocytes [220,267,268]. Its cytotoxic action stems from catalyzing the reaction between hydrogen peroxide and chloride in the extracellular medium, resulting in the formation of hypochlorous acid, a highly oxidizing species effective in eliminating bacteria, fungi, and various other pathogenic microorganisms [220,267,268]. Furthermore, it has been linked to autoimmune inflammatory diseases due to its direct association with neutrophils and their role in inflammatory mechanisms. Its role in IBD has been extensively studied, with fecal levels of this enzyme correlating with increased severity of the pathology [220,241,242]. Additionally, its presence in intestinal mucosa can also be utilized for monitoring purposes in patients undergoing treatment for UC. Moreover, the increased presence of 3-chlorotyrosine (3-Cl-Tyr), a modification product obtained through the reaction of hypochlorous acid with tyrosine residues, has been observed in the colons of IBD patients [242,268]. Given the above explanation, investigating MPO as a biomarker in IBD holds promise for clinical utility.

Matrix metalloproteinases (MMPs): Human MMPs constitute a group of 24 zinc-dependent endopeptidases classified according to their substrate preference and domain structure into collagenases, gelatinases, stromelysins, and membrane-type MMPs. MMPs are involved in various processes, including extracellular matrix processing and facilitating the migration of inflammatory mediators to sites of damage [243,269,270].

Studies have revealed an increase in the levels of MMPs, specifically MMP1, MMP2, MMP9, and MMP13, in the intestinal mucosa and faces of patients with IBD compared to healthy individuals [220,243]. This increase is particularly observed in the inflamed mucosa of patients with IBD, both UC and CD, in contrast to healthy individuals and the non-inflamed mucosa of IBD patients [220,243].

Furthermore, an elevation in MMP9 levels has been observed in the sera of patients with active UC and CD compared to healthy subjects and inactive CD and UC [271]. Additionally, fecal MMP9 levels have shown high sensitivity and specificity in identifying UC patients with endoscopic activity, although the values obtained for CD were not sufficiently accurate for diagnosing this disease [220].

Other fecal biomarkers: Numerous other fecal markers have been studied for IBD, including polymorphonuclear neutrophil (PMN)-elastase [232], neutrophil gelatinase-associated lipocalin (NGAL) (also known as lipocalin-2) [220,241], intestinal alkaline phosphatase (IAP) [220], and M2-pyruvate kinase (M2-PK) [244], among others.

Other frequently used biomarkers include the fecal immunochemical test (FIT) and urinary prostaglandin E-major urinary metabolite (PGE-MUM) [216].

### 3.4. Biomarker Evaluation Techniques

The primary method of protein detection is the enzyme-linked immunosorbent assay (ELISA). It is an enzyme immunoassay that employs a capture antibody to bind the biomarker and a specific detection antibody to recognize it. This recognition event enables the detection of the bound biomarker using a conjugated enzyme, which catalyzes a reaction generating a measurable product. The quantity of this product correlates with the concentration of the biomarker in the sample. Additional common techniques for protein detection include fluorescent-based immunoassays (FIAs), radioimmunoassay (RIA), Western blot (WB), and mass spectrometry (MR) [213]. FIA and RIA working principles are similar to ELISA but differ in the label used, as RIA employs radiolabels and FIA fluorescent compounds. Western blot separates protein homogenates through electrophoresis to subsequently transfer onto a nitrocellulose or nylon membrane through electro-blotting and, finally, incubate with labeled antibodies to quantify the obtained bands of proteins or detect a particular antigen [272]. Lastly, MS is based on protein identification and quantification based on their mass-to-charge ratio (*m*/*z*). For this purpose, proteins are enzymatically digested, ionized, and accelerated and its mass spectrum is defined. This mass spectrum is analyzed and matched through a search algorithm to determine the proteins present in the sample and their quantity. Additionally, it is a versatile method that can be used for other biomolecules like RNA, DNA, and lipids [273,274].

Besides proteins, biomarkers also encompass DNA and RNA, which are invaluable molecules for study. Polymerase chain reaction (PCR) stands out as the primary technique for analyzing DNA and RNA, with variations like reverse transcription–PCR and real-time PCR offering diverse applications. Additionally, northern blot and DNA microarray methods are also employed for this purpose [213]. Notably, microbiota analysis often involves studying 16S RNA in prokaryotic and 18S RNA in eukaryotic microorganisms, which is particularly important when microorganisms cannot be grown in specific culture media [143].

Despite many other techniques under development, such as cell membrane microarray [98], protein microarray [275,276], or sensors [215,277], the aforementioned methods are frequently utilized in hospital settings. Microarray technology is based on the immobilization of certain molecules (ssDNA, ssRNA, proteins, or cell membrane homogenates) on a surface in order to detect the biomarkers of the sample according to the specific recognition between them and the immobilized molecule. Notably, cell membrane microarrays enable personalized medicine by allowing for the immobilization and longitudinal study of a patient’s own biopsies [95]. For genetic biomarker detection, DNA microarrays usually label DNA or RNA samples with fluorescent ligands, and in protein and cell membrane microarrays, conjugated detection antibodies are employed [98,278].

## 4. Therapeutic Targets and Treatments in IBD

As IBD has no cure yet, its therapeutic options are mainly based on the control of inflammatory symptoms [14,279]. Treatment options include corticosteroids, aminosalicylates, immunosuppressants, and biological drugs [14,15,21].

Aminosalicylates, such as 5-aminosalicylic (5-ASA), present multiple anti-inflammatory and immunomodulatory actions [14,279,280]. They inhibit lipoxygenase and cyclooxygenase (interfering in the generation of pro-inflammatory products), remove reactive oxygen species, prevent the recruitment of immune cells and cytokine formation, and induce Treg through TGF-β anti-inflammatory cytokine production [14,280]. They serve as a primary treatment option for UC patients with mild to moderate disease [280]. In the same line, corticosteroids (CSs), like budesonide, decrease the anomalous immune response, reducing the pro-inflammatory cytokines’ release [14,279,280]. Hence, they act as broad-spectrum systemic anti-inflammatory therapies that achieve the control of inflammation. They are effective for moderate to severe UC and CD patients during short-term treatments, as long-term treatment generates undesirable adverse effects. Nonetheless, second-generation CSs have been developed in order to reduce these adverse effects [280].

Other immunomodulators, such as thiopurines or methotrexate, interfere with the biosynthesis of nucleosides or are directly analogous to them, damaging the DNA of the cells and specifically inhibiting T cell proliferation and pro-inflammatory cytokines in IBD [14,280,281]. They are used when CSs or aminosalicylates do not work or along with anti-TNF treatment to reduce the production of anti-biological antibodies [280].

Other types of therapeutical options are biological drugs, also known as biologics, which are antibodies directed against pro-inflammatory cytokines TNF (infliximab or adalimumab), IL-12 (ustekinumab), and IL-23 (ustekinumab, risankizumab, or mirikizumab) or inhibitors of integrin (vedolizumab, natalizumab, or etrolizumab), a glycoprotein receptor of the cell surfaces [14,279,282]. Biologics targeting integrins prevent leukocytes from infiltrating the intestine. Therefore, anti-integrins, along with those that target pro-inflammatory cytokines, contribute to reducing the inflammatory response in the intestine [14,67]. They aid many IBD patients who are unresponsive to conventional anti-inflammatory and immunomodulator therapies. These medications are more specific than conventional ones targeting specific cytokines or receptors that cause the characteristic inflammation. Nowadays, they have been used as a primary treatment in specific clinical scenarios [280].

The next developed therapeutic groups were small-molecule drugs (SMDs), being the next generation of immunomodulators. They are exemplified by tofacitinib, an oral small-molecule Janus kinase (JAK) inhibitor. Regarding tofacitinib, it inhibits JAK-1, JAK-2, and JAK-3; consequently, gamma-chain-containing cytokines are also inhibited [14,280,283]. It is also effective in blocking T cells and NK cells, as well as modulating pro-inflammatory cytokines [280].

Additionally, because the microbiota is an essential part of IBD onset, the use of antibiotics, probiotics, prebiotics, synbiotics, and postbiotics is a useful novel therapy, along with fecal microbiota transplantation (FMT) [14]. Antimicrobials are used to eliminate pathogenic invasion, but they should be short-termed, as they produce resistance, in addition to the possible use of phage therapy [13,33,280]. However, probiotics, prebiotics, synbiotics, postbiotics, and FMT support the improvement of the microbiota, achieving the maintenance of the commensal microbiota [14,33]. Therefore, while microbiota beneficial effects can be restored in IBD patients using those strategies, the specific relationship between beneficial and harmful microbiotas is still being researched, making these approaches less effective than traditional therapies.

Finally, some other options to manage the disease are not based on medications, such as surgical interventions [13,14], nutritional treatment [283,284,285], botanical treatment [13], stem cell transplantation [14], and apheresis treatment [14], among others.

Regarding EIM treatment, the European Crohn’s and Colitis Organisation (ECCO) made a guideline for standardizing them [286]. One out of two patients suffer from EIMs at some point, meaning these symptoms must also be considered in their treatment. Additionally, manifestations, such as primary sclerosing cholangitis (PSC) or venous thromboembolic events (VTE), are associated with high mortality [286].

For the treatment of VTE, the use of direct oral anticoagulants is recommended, as well as preventive treatment in patients hospitalized due to acute medical illness or major surgery with heparin or fondaparinux. For the treatment of PSC, ursodeoxycholic acid is recommended to improve liver biochemistry, although its effect on the disease itself is unknown [286].

Treatment for other EIMs is also important due to their morbidity. For instance, IBD-related arthritis is managed with NSAIDs or cyclooxygenase-2 (COX-2)-specific inhibitors for short-term treatment. Oral aphthous ulcers are recommended to be treated with supportive mouth care, including topical steroids, along with the treatment of IBD itself. Pyoderma gangrenosum is suggested to be treated using anti-TNF biologics, specifically infliximab. It can also be treated with systemic and topical steroids, ciclosporin, ustekinumab, dapsone, metronidazole, topical calcineurin inhibitors, and tetracyclines. Anemia, the most common EIM of IBD, is managed by determining the etiology of the condition and treating the underlying deficiency or pathology. For example, iron deficiency anemia is treated with oral and intravenous iron, and megaloblastic anemia is treated with vitamin B12 and folate supplementation [286].

However, some EIMs require more complex treatment. For instance, fatigue is an intricate symptom to treat, as its etiology in IBD is still under research. Therefore, physicians should investigate the underlying altered mechanisms to treat them effectively. Likewise, psychosocial interventions and anti-TNF biologicals are recognized as useful tools for reducing fatigue, whereas CSs and immunomodulators may exacerbate it [286].

## 5. Current Challenges

After assessing the primary molecular bases, biomarkers, and available therapeutic options for IBD, a SWOT analysis (Strengths, Weaknesses, Opportunities, and Threats) was conducted (Table 3) to evaluate the present state of managing this condition.

From this evaluation, the main challenges encountered lie in the unclear definition of specific factors that trigger IBD. Multiple interconnected elements from the genetics, immune system, microbiota, and environment contribute to the development of IBD. Consequently, there is no specific therapeutic option to cure this disorder, leading to the prescription of symptomatic treatments. However, these treatments frequently result in non-response or resistance, particularly with biological medications where anti-biologic antibodies can be developed. Continuous monitoring of therapeutic response and resistance is urgently needed to adjust treatment strategies and achieve disease remission, alongside the development of more potent therapies.

While the biomarkers’ follow-up relies on the ELISA technique as the gold-standard method, it has inherent limitations. One major hurdle is its lack of individualized and personalized configuration. ELISA assays typically utilize a 96-well plate configuration, which, while cost-effective for analyzing numerous samples simultaneously, can present challenges during acute disease flare-ups. In such instances, patients may face delays in accessing crucial diagnostic information as they await the accumulation of a sufficient number of samples for testing to occur.

In light of these limitations, novel techniques that overcome these challenges by offering faster, simpler, individualized, cost-effective solutions with improved sensitivity have been proposed. Microarray is a polyvalent technology that meets all of the mentioned characteristics, as it allows for the immobilization of proteins (such as antibodies [275,287], cytokines, and microbial protein extracts [276]) and cell membranes [98], enabling the development of a comprehensive, robust, small, sensitive, and customized panel of biomarkers. This technique enables the simultaneous evaluation of multiple biomarkers, while also being adaptable to point-of-care (POC) methodologies. Another potential method for POC devices is giant magnetoresistant (GMR) biosensors. These sensors offer versatility in configuration, leading to the development of multiple types of GMR sensors tailored to fit the specific aim of biomedical applications. Among these, flexible GMR sensors stand out for their wearable biosensing option [288].

Because biomarkers rise in the time before disease remission occurs, methods that detect them in real time would aid in the prognosis of the disease by controlling and alleviating the predicted symptoms. For this purpose, a small sensor device can be useful in continuous detection for anticipating the biological events that trigger inflammation. For instance, screen-printed electrodes (SPEs) based on electrochemical reactions [289] or temperature sensors [277] have been developed. Nonetheless, it is crucial to maintain real-time monitoring for clinical applications. The complexity of empowering patients to manage their own disease and treatment is particularly evident in conditions like IBD, where the underlying causes remain elusive. There is still much to learn in both basic and applied knowledge of IBD, and progress will likely continue for many years to come.

In addition to these assays, clinical tools are an essential part of IBD management. In this sense, imaging techniques are also vital in acquiring relevant information, including, for example, plain radiography, barium contrast studies, ultrasonography, CT, or MRI [290]. Among these, ultrasound stands out for its high accuracy in diagnosis, disease evaluation, and monitoring. It is a non-invasive and cost-effective technique that does not require specific preparation, and it can be used as a POC device. Hence, ultrasounds could replace other clinical tools that are invasive, expensive, and complicated, such as CT or MRI [291]. Likewise, this tool is capable of being applied to primary care settings. Nonetheless, those clinical approaches are often reserved for specialized care settings. Consequently, primary care professionals lack helpful tools that aid in IBD management, which hinders the diagnosis, prognosis, and monitoring of IBD, ultimately decreasing the well-being of patients as the diagnosis is delayed and their symptomatology is not correctly controlled until the patient arrives at the specialist [20,292]. Additionally, these delays in diagnosis seem to increase the probability of developing a more complicated form of the disease [20].

Therefore, improving diagnosis, biomarker detection, and monitoring would enhance the quality of life of patients who are usually constantly suffering from flare-ups owing to incorrect treatment dosage, among other causes [293]. However, there is still a lack of concern about patients’ perspectives regarding treatment. For instance, treatment is usually focused on inflammatory symptoms, while the main concerns of the patients are often the psychosocial symptoms, such as fatigue, which are usually more debilitating for them than intestinal ones [294]. Moreover, these symptoms are still unclear and not fully understood by health professionals, being less studied even though they are already associated with a lower quality of life and psychological well-being, along with other issues, such as pain or anemia [294]. In short, addressing the unmet treatment needs of patients is essential for ultimately improving their quality of life.

In conclusion, researchers typically focus on improving diagnostic tools and treatments to advance the clinical field of IBD, which becomes highly valuable when their innovations are finally at the disposal of physicians. However, they may sometimes overlook the real concerns and unmet needs of patients. Often, patients do not necessarily need new and sophisticated diagnostic and prognostic methods, as their main symptoms are already managed effectively with current approaches. Instead, they require specific attention to the overlooked manifestations of the disease, which can be more debilitating than the principal symptoms.

## Figures and Tables

**Table 1:** Loci, gene, function, and associated disease of each genetic determinant related to IBD.

Loci	Gene	Function	Associated Disease	References
IBD1	NOD2/CARD15	Microorganism detection	CD	[33,38,40,48,50,52,53,54,76,80,81]
IBD3	HLA class I	Autotolerance	IBD	[12,37,38,49,57,58,59,60,61,62,63,64,65,66,67,68,69,70,71]
	HLA class II			
	HLA class III	Triggers inflammatory response; stimulates antigen uptake		
IBD5	IRF1	Transcription factor that stimulates pro-inflammatory cytokines;	IBD	[75,76,77,86]
	SLC22A4	L-carnitine transporter	IBD (CD)	
	SLC22A5			
	IL-10; IL-10R	Anti-inflammatory response	IBD	[38,78,79]
	IL-23; IL-23R	Pro-inflammatory response	IBD	[38,40]
	IL1RA	IL-1 receptor antagonist	Pediatric IBD	[38,80]
	ATG16L1	Autophagy	CD	[38,81]
	IRGM	Autophagy	CD	[38,81]
	PTPN2	Autophagy	IBD	[38,80]
	CDH1	E-cadherin of adherent junction production	UC	[38]
	HNF4-α	Expression of cell junctions	UC	[38,40]
	ULK1	Autophagy	IBD	[43]
	IL8RA	Pro-inflammatory response	EIMs in IBD	[4,95]
	PRDM1	Regulation of immune response	EIMs in IBD	[4,95]
	USP15	Deubiquitination of proteins	EIMs in IBD	[4,95]
	TIMP3	Anti-inflammatory response	EIMs in IBD	[4,95]
	ITGB3	Component of certain integrin receptors	EIMs in IBD	[4,95]
	SOCS5	Anti-inflammatory response	EIMs in IBD	[4,95]
	CLEC4K/CD207	Innate immune response	EIMs in IBD	[4,95]
	ITGAL	Component of integrins	EIMs in IBD	[4,95]
	PTGER4	Prostaglandin E2 receptor	EIMs in IBD	[4,95]
	TYK2	Cytokine receptor	EIMs in IBD	[4,95]
	STAT3	Transcription factor of cytokines and growth factors	EIMs in IBD	[4,95]
	JAK2	Cytokine and growth factor signaling	EIMs in IBD	[4,95]
	SOCS1	Suppressor of cytokine signaling	EIMs in IBD	[4,95]
	FOXO1	Myogenic growth and differentiation	EIMs in IBD	[4,95]
	IRF8	Regulation of genes involved in immune response	EIMs in IBD	[4,95]
	BCL211	Apoptotic activator	EIMs in IBD	[4,95]
	UBASH3A	Modulates T cell activation and function	EIMs in IBD	[4,95]
	ACADM	Degradation of medium-chain fatty acids	UC	[83]
	PDK1	Regulation of glucose and fatty acid metabolisms	UC	[83]
	FIS1	Mitochondrial fission	UC	[83]
	LACC1	Purine nucleoside enzyme regulating redox balance and preventing cytoplasmic acidification	CD	[85,87]
	GPX1; GPX3	Reduce organic peroxide and hydrogen peroxide	CD	[85,88]
	ALDH2	Cellular metabolisms	IBD	[64,85]
	STAT3	Transcription factor	IBD	[85,89]
	PARK7	Redox sensing	IBD	[83,85]
	LRRK2	Autophagy, mitophagy, apoptosis	CD	[85,91,94]
	GAK	Clathrin-coated vesicle trafficking	IBD	[90,94]
	MAPT	Microtubules and axonal transport	IBD	[90,94]

**Table 2:** Certain inflammatory bowel disease biomarkers, classification, and associated disease.

Classification		Biomarker	Associated Disease	References
Genetic and epigenetic biomarkers		NOD2; PRDM1; NDP52	CD	
		KIF9-AS1; LINC01272; DIO3OS; DQ786243; CDKN2B-AS1 (ANRIL); IFNG-AS	IBD	[218,219]
		miR-21; miR-223; miR-155	IBD	[220,221,222]
		miR-375	UC	[223,224]
Blood Biomarkers	Serological biomarkers	perinuclear antineutrophil cytoplasmic antibodies (pANCA)	UC	[220,225]
		Anti-*Saccharomyces cerevisiae* antibodies (ASCA)	CD	[220,226,227]
		C reactive protein (CRP)	IBD	[220,226,228]
		Pro-inflammatory cytokines (TNF, IL-1β, IL-12, IL,23, etc.)	IBD	[62,67,215,220,229,230]
		Anti-OmpC	CD	[220,228,231]
		Pancreatic antibodies (PABs)	CD	[231,232]
		Anti-carbohydrate antibodies (ALCA, ACCA, AMCA)	CD	[231,233,234,235]
		Cytokine oncostatin M (OSM)	IBD	[228,236]
		Antibodies anti-membrane antigens	IBD	[98]
		Leucine-rich α2 glycoprotein (LRG)	IBD	[214,223]
	Hematological parameters	Erythrocyte sedimentation rate (ESR); total white blood cell (WBC); eosinophil (EOS) count; platelet (PLT) count	IBD	[223,237]
Fecal biomarkers		Calprotectin	IBD	[220,223,226]
		Lactoferrin	IBD	[158,220,238,239]
		FIT	IBD	[216]
		Calgranulin C (S100A12)	IBD	[158,238,240]
		Myeloperoxidase (MPO)	IBD	[220,241,242]
		Matrix metalloproteinases (MMPs)	IBD	[220,243]

**Table 3:** SWOT analysis of the current state of IBD management.

**Weaknesses**	**Strengths**
Unknown etiology Relapses and remission of the disease Lack of specific biomarkers to determine clinical severity of the disease Difficulty in diagnosing disease subtypes Lack of early detection of flare-ups Absence of real-time monitoring High-cost monitoring methods Lack of curative treatment Variability of responses to IBD treatments Treatment resistance and loss of response No concise therapeutic aim Lack of sequential therapy strategy	Effective monitoring through ELISA Specific treatment options available Novel therapeutic strategies under development Introduction of wearable sensors for real-time monitoring Personalized diagnostic methods under development New pharmacological treatments Artificial intelligence improves IBD diagnosis and patient care management Digitalization allows for remote monitoring of patient’s treatment
**Threats**	**Opportunities**
Potential for misinterpretation of biomarker data Uncertainty regarding the long-term efficacy and safety of new treatments Regulatory challenges in approving new treatments Limited funding for research and development Challenges in educating healthcare professionals and patients about new advancements Lack of patient perspective Treatment of inflammation only	Exploration of the disease’s etiology to identify new treatment targets Identification of new specific biomarkers for diagnosis and patient stratification Development of real-time monitoring technologies Identification of cost-effective monitoring methods Strategies to minimize relapses and maintain remission Personalized treatment approaches Potential for biosimilar self-administration Efficiency analysis Comparison of new drug compounds Strategies with new drugs
